# Effects of Food Consumption on Self-rated Health: Evidence from Korean Adolescents

**Published:** 2018-07

**Authors:** Ee-Gyeong KIM, Sarah CHUNG, Min Gyo CHUNG

**Affiliations:** 1. Dept. of Education, Chung-Ang University, Seoul, Korea; 2. Harris School of Public Policy, University of Chicago, Chicago, IL, USA; 3. Dept. of Computer Science, Seoul Women’s University, Seoul, Korea

## Dear Editor-in-Chief

Self-rated health (SRH) refers to the self-rating of an individual’s overall health status. Inherently, SRH involves subjective judgment, so some studies threw doubts on its reliability. However, SRH was a valid indicator of people’s health status and has been widely used to measure and monitor health status of young people, middle-aged population, and the elderly people. Much has been reported of the factors having effects on SRH. SRH was found to have statistically significant relationships with personality characteristics such as agreeableness, extraversion, conscientiousness, and neuroticism (1), and socio-economic status, lifestyle, and psychosocial factors (2). Meanwhile, food consumption was one of the important variables that attracted attention in public health study, so there have been many studies related to food consumption (3, 4). However, little is known about how food consumption correlates with SRH. In this study, therefore, we explored the relationships between food consumption and SRH, using a representative sample of Korean adolescents. Specifically, we took into account 9 foods, classified them into unhealthy foods (called U-foods) and healthy foods (called H-foods), and probed into how U-foods intake and H-foods intake have their respective effects on SRH.

The present study used the data from the 11^th^ KYRBS (Korea Youth Risk Behavior Web-based Survey), conducted in 2015 (5). KYRBS is a nationwide self-administered online survey to identify the health behaviors of Korean secondary school students. KYRBS sampled 68043 students over 797 schools. SRH was measured by asking students the question “What do you usually think about your health condition?” For binary logistic regression, the responses were dichotomized. We selected 9 foods: 6 foods (carbonated drinks, high caffeine drinks, sweet drinks, fast food, “ramen” Korean noodles, and confectionery) were labeled as U-foods, while 3 foods (fruits, vegetables, and milk) were labeled as H-foods. Multivariable logistic regression analyses were used to investigate the association between food consumption and SRH. The significance level of all the hypothesis tests was set at *P*<0.05.

The present study revealed the association between U-foods/H-foods consumption and SRH (Table 1). The increased consumption of 3 H-foods was positively associated with “good” SRH, which indicates that Korean students had the notion that frequent intake of H-foods would ultimately benefit their health. A high daily intake of fruits and vegetables promoted health and prevented chronic diseases, and that milk consumption was inversely associated with the risk of cardiovascular disease. In case of U-foods, the consumption of high caffeine drinks, sweet drinks, and fast food more than 5 times/week were inversely associated with “good” SRH.

**Table 1:** Adjusted odds ratios (AORs) for “good” SRH (vs. reference category “bad”) according to frequency of eating 6 U-foods and 3 H-foods (reference category “never”)

***Variables***	***AOR (95% CI)***	***P-value***	***Variables***	***AOR (95% CI)***	***P-value***
***Carbonated drinks***	***6 U-foods***				
	***High caffeine drinks***				
≥ 5times/week	.936(.862–1.016)	.113	≥ 5times/week	.638(.544–.747)	.000
3–4times/week	1.044(.980–1.113)	.183	3–4times/week	.899(.784–1.031)	.127
1–2times/week	1.077(1.026–1.131)	.003	1–2times/week	.956(.896–1.020)	.173
Never Sweet drinks	1		Never Fast food	1	
≥ 5times/week	.906(.839–.978)	.011	≥ 5times/week	.872(.770–.987)	.031
3–4times/week	1.026(.959–1.097)	.462	3–4times/week	.877(.820–.937)	.000
1–2times/week	1.032(.974–1.093)	.286	1–2times/week	.993(.948–1.040)	.759
Never Ramen noodles	1		Never Confectionery	1	
≥ 5times/week	.965(.874–1.065)	.476	≥ 5times/week	.987(.915–1.064)	.730
3–4times/week	1.098(1.033–1.167)	.003	3–4times/week	1.024(.963–1.089)	.445
1–2times/week	1.074(1.025–1.125)	.003	1–2times/week	1.065(1.010–1.122)	.019
Never Fruits	1		Never Vegetables	1	
	3 H-foods				
≥ 5times/week	1.186(1.102–1.276)	.000	≥ 5times/week	1.537(1.403–1.684)	.000
3–4times/week	1.224(1.135–1.321)	.000	3–4times/week	1.302(1.188–1.428)	.000
1–2times/week	1.171(1.089–1.260)	.000	1–2times/week	1.176(1.071–1.293)	.001
Never Milk	1		Never	1	
≥ 5times/week	1.176(1.113–1.242)	.000			
3–4times/week	1.158(1.091–1.230)	.000			
1–2times/week	1.132(1.065–1.203)	.000			
Never	1				

Students’ health perception toward the above 3 U-foods can be explained by earlier studies asserting that caffeine was the commonly used psychoactive substance and frequent consumption of high caffeine drinks likely led to acute visual loss, palpitations, or ventricular arrhythmia; sweet drinks had a positive association with obesity risk; and fast food was a major factor in sodium intake, significantly associated with the risk of obesity, cardiovascular disease, or gastrointestinal disorder. To the contrary, the consumption of the 3 U-foods (i.e., carbonated drinks, ramen noodles, and confectionery) 1∼2 times/week was positively associated with “good” SRH. Carbonated drinks were associated with periodontal disease, coronary artery calcification, or chronic kidney disease; ramen noodles were identified to induce gastroesophageal reflux symptom; and confectionery products, usually containing high amount of carbohydrate, were likely to raise diabetes risk and to have long-term effects on adult violence. However, Korean adolescents tended to think that a little intake of carbonated drinks, ramen noodles, and confectionery, even though they were known to be unhealthy foods, did not matter to their health. This raises a concern that Korean adolescents underestimate the influence of these U-foods over their health. To remedy this misperception, school-based interventions should be considered to help Korean adolescents to shift away from unhealthy foods and choose healthy foods for their future health and well-being.
